# Child Welfare in India

**Published:** 1920-04-17

**Authors:** 


THE PUBLIC WELL-BEING—(*continued*)

### Child Welfare in India.

A FEATURE of the Maternity and Infant-Welfare Exhibition which was held at Delhi last month was exhibits by employers of labour showing what they have done to improve the conditions of their workpeople. There were also models of Indian houses, illustrating first unhygienic dwellings and then dwellings as they ought to be. A silver medal was awarded for the best model plan of a house suited to, first, an Indian family whose income is Rs.30 per month, secondly to a family whose income is more than that amount or less than Rs.100 per month. Lectures were delivered by medical experts, with the assistance of magic-lanterns and cinema films, on infant feeding and rearing, kindergarten training, the psychology of childhood, the care and training of mentally deficient children, domestic science, disease, flies, plague, malaria, food. It is hoped that the exhibition will be the beginning of a widespread campaign having for its object the reduction of the terrible maternal and infant mortality that prevails in India. At present out of every 1,000 children born 250 die before they reach the age of one year, and it is notorious that a large proportion of these deaths are due to preventable causes. The field of work here for educated Indians is unlimited, and if adequate co-operation is given to the movement which is being set on foot the effect on the health and prosperity of the country will be of incalculable value.

